# A Wearable System for Knee Osteoarthritis: Based on Multimodal Physiological Signal Assessment and Intelligent Rehabilitation

**DOI:** 10.3390/s25237334

**Published:** 2025-12-02

**Authors:** Jingyi Hu, Shuyi Wang, Yichun Shen, Xinrong Miao

**Affiliations:** 1 School of Health Science and Engineering, University of Shanghai for Science and Technology, Shanghai 200093, China; 2 Institute of Robotics and Intelligent System, Dalian University of Technology, Dalian 116024, China

**Keywords:** knee osteoarthritis (KOA), gait analysis, surface electromyography (sEMG), intelligent rehabilitation, wearable system

## Abstract

Knee osteoarthritis (KOA), a common degenerative joint disease, affects a large patient population and poses significant challenges in early diagnosis and rehabilitation. Achieving precise assessment of knee function and efficient home-based intelligent rehabilitation is crucial for alleviating pain, slowing disease progression, and improving patients’ quality of life. This study proposes a smart wearable knee function assessment based on multimodal physiological signals and a rehabilitation system. The system integrates surface electromyography (sEMG), pressure sensors, and an inertial measurement unit (IMU) to synchronously capture gait, posture, and muscle activity. It quantifies knee function by extracting gait and EMG features. Additionally, a wearable massage device driven by airbags was designed and implemented to simulate the traditional Chinese medicine “seated knee-adjustment method” and deliver precise intelligent rehabilitation interventions. Experimental results validated the system’s accuracy in functional assessment and reliability in rehabilitation assistance. The average relative error in gait feature extraction was below 8%, while the massage head displacement error remained within clinically acceptable ranges. By integrating multimodal sensing technology with intelligent rehabilitation devices, this system offers KOA patients a convenient, efficient, and sustainable home-based rehabilitation solution with strong clinical application potential and promotional value.

## 1. Introduction

Knee osteoarthritis (KOA) is a chronic degenerative disease that significantly impacts the quality of life in middle-aged and elderly individuals. Characterized by pathological changes such as articular cartilage degeneration, subchondral bone remodeling, and synovitis, it is a major cause of pain and functional limitations in older adults [1,2]. According to the latest analysis from the Global Burden of Disease (GBD) 2021 study, the global prevalence and disability-adjusted life years (DALYs) for KOA continue to rise, with China also showing a significant upward trend in age-standardized incidence and prevalence rates [3]. A systematic review and meta-analysis of the Chinese population from 2013 to 2023 revealed an overall KOA prevalence of 28.0%, with 33.9% among women and 20.5% among men. The highest prevalence was observed in the 60–69 age group (32.8%) [4]. The convergence of population aging and risk factors such as obesity not only severely compromises the quality of life for the elderly but also imposes a persistent and substantial economic burden on families and healthcare systems.

The clinical diagnosis of KOA currently relies primarily on a comprehensive assessment of symptoms, physical signs, and imaging studies [5]. Clinically, pain, stiffness, and limitations in daily activities are often evaluated using the Visual Analogue Scale (VAS) and the Western Ontario and McMaster Universities Arthritis Index (WOMAC). These methods have been recommended by multiple guidelines as standardized measurement tools [6,7,8]. In radiology, X-ray Kellgren–Lawrence (K–L) grading remains the most widely used evaluation standard. Magnetic resonance imaging (MRI), however, can be combined with semi-quantitative scoring systems such as Recht to provide detailed assessments of structures like cartilage, menisci, and synovium [9,10,11]. However, this traditional diagnostic pathway faces limitations: it heavily relies on patient subjective reports and physician experience, leading to issues such as low inter-observer consistency and potential overlooking of early symptoms. Furthermore, radiographic changes often lag behind early biomechanical and cartilage microstructural damage. By the time definitive imaging progression is evident, many patients’ lesions have become irreversible, and the optimal window for intervention has often been missed.

To compensate for the subjectivity and lag associated with traditional diagnostic methods, numerous studies in recent years have introduced deep learning methods for automated grading of KOA imaging. Aleksei Tiulpin proposed a KOA auto-grading method based on Deep Siamese Convolutional Neural Networks (CNNs), validated on large public datasets like the Osteoarthritis Initiative (OAI). Results demonstrated high consistency (Kappa = 0.83) and excellent accuracy (AUC = 0.93) across multi-classification tasks [12]. Wang Yifan et al. combined You Only Look Once (YOLO) object detection with visual Transformers to propose an end-to-end KOA auto-diagnosis method. It achieved 95.57% segmentation accuracy on large-scale datasets and improved accuracy by 2.5% over traditional CNNs in grading tasks [13]. Overall, these studies significantly enhance the objectivity and automation of KOA imaging grading. However, they remain primarily focused on “offline interpretation of static images,” making it challenging to capture dynamic load variations and functional states during real-world activities. Consequently, their support for developing personalized rehabilitation strategies remains limited.

To further overcome these limitations, it is necessary to re-examine KOA from the perspectives of pathogenesis and lower limb biomechanics. Previous studies indicate that KOA results not only from localized joint tissue degeneration but also reflects overall lower limb biomechanical imbalance: multiple factors such as decreased muscle strength, altered neuromuscular control strategies, and abnormal joint kinematics and dynamics interact synergistically to drive disease progression [14]. Early studies revealed that surface electromyography (sEMG) activation patterns in muscle groups like the quadriceps during walking significantly differed between KOA patients and healthy individuals. These differences manifested as altered synergistic patterns and increased muscle co-contraction, with such neuromuscular characteristics detectable even before significant radiographic changes appear [15]. Further studies indicate that pain anticipation and fear-avoidance beliefs correlate significantly with co-contraction levels in muscles surrounding the knee joint, suggesting central regulation and psychological factors also contribute to the development of KOA gait abnormalities [16]. Concurrently, wearable gait monitoring technology based on inertial measurement units (IMUs) has advanced rapidly: systematic reviews demonstrate that IMUs placed on the lumbar spine, lower leg, and other sites can reliably quantify spatiotemporal gait parameters, joint angles, and acceleration characteristics in KOA patients during real-life environments, providing novel tools for objective functional assessment [17]. Early studies have also validated the advantages of wearable sensor-based gait analysis in terms of cost, scenario flexibility, and long-term follow-up [18]. Thus, establishing an objective assessment framework for KOA at both the “neuromuscular control” and “kinematics and dynamics” levels possesses a clear theoretical and technical foundation.

Against this backdrop, a practical approach to achieving continuous, objective assessment of disease progression and functional status in KOA patients involves integrating wearable gait monitoring with muscle function quantification to establish a sensor-based motion-fatigue evaluation system. Regarding muscle fatigue assessment, sEMG frequency-domain features such as mean power frequency (MPF), spectral median frequency (MF) and zero-crossing rate (ZCR) have been extensively employed to reflect muscle fiber recruitment and metabolic status, with systematic application in exercise fatigue research [19]. Classic studies indicate that as local muscle fatigue develops, the MF of the sEMG spectrum gradually decreases and the ZCR undergoes regular changes, serving as sensitive indicators of fatigue accumulation during prolonged tasks [20,21]. Building upon this, further research has proposed combining parameters such as ZCR and amplitude to construct composite fatigue indices, utilizing machine learning algorithms for automatic recognition of muscle fatigue during movement [22,23]. These findings suggest that jointly modeling electromyographic fatigue indicators with IMU-based spatiotemporal gait parameters may offer a more comprehensive characterization of the “function–load–fatigue” coupling in KOA patients during actual activities, moving beyond limitations of static muscle strength or subjective pain ratings.

As a degenerative disease, KOA involves damaged structures that are difficult to reverse. Therefore, the overarching principles for KOA treatment are pain control, improvement of joint function, and enhancement of patient quality of life [24]. During the rehabilitation phase, tailored treatment approaches are employed based on individual patient conditions. Currently, common treatment modalities encompass four categories: basic therapy, medication, restorative therapy, and reconstructive therapy. While medication can alleviate symptoms, it may carry certain adverse effects. Restorative and reconstructive therapies often require extended rehabilitation periods and are typically reserved for advanced-stage patients, generally not serving as first-line options [25]. In contrast, foundational therapies such as physical therapy and massage offer the advantages of safety and non-invasiveness, making them more suitable for early-stage patients. However, their implementation relies heavily on practitioner experience, posing challenges for quantifiable and standardized treatment delivery.

Parallel to biomechanical assessments, wearable lower-limb robotic and soft exoskeleton technologies have been advancing. Recent reviews indicate that lower-limb rehabilitation exoskeletons can replace partial muscle function through rigid or compliant actuation, providing assistance and training for daily activities such as walking, standing-to-sitting transitions, and stair climbing. However, most systems remain at the prototype or small-scale clinical trial stage, with limited commercialization and home-based applications [26,27]. Zhu et al. designed an integrated multi-joint lower-limb rehabilitation exoskeleton encompassing hip, knee, and ankle joints, achieving closed-loop joint position control and trajectory planning, thereby establishing an engineering foundation for complex multi-joint coordinated control [28]. In studies targeting KOA populations, McGibbon et al. evaluated the Keeogo™ knee power exoskeleton in clinical training and home-use settings. Results demonstrated that long-term training and home use significantly improved stair-climbing ability and reduced WOMAC pain and functional scores in KOA patients [29]. Conversely, home-based neuromuscular electrical stimulation (NMES) has proven effective as an alternative or supplement to traditional resistance training. Randomized controlled trials indicate that mobile app-based NMES systems significantly improve quadriceps muscle cross-sectional area and functional performance in moderate-to-severe KOA patients, demonstrating good safety and efficacy in reducing pain, stiffness, and enhancing function [30,31]. These findings indicate that integrating “wearable aids” with “home-based interventions” represents a key direction for advancing precision rehabilitation for KOA from hospital settings into daily life.

Despite significant advances in applying sensing technology and wearable robotics to KOA diagnosis and rehabilitation, current research still faces several critical limitations. First, most biomechanical and machine learning studies focus separately on diagnosis or functional assessment, predominantly using short-term laboratory gait or static imaging data for offline analysis. There remains a lack of closed-loop systems for long-term, continuous monitoring of KOA patients in real-life scenarios, coupled with intervention strategies. Second, existing wearable devices often lack a closed-loop “assessment-intervention-feedback” control architecture centered on core feedback variables such as knee joint load, muscle activation, and fatigue status. Third, existing exoskeletons face challenges in comfort, weight, convenience, and long-term compliance, making it difficult to fully meet the routine usage needs of early-to-mid-stage KOA patients in home and community settings.

In summary, this study developed a multimodal physiological signal assessment and intelligent wearable rehabilitation system for KOA patients. By deeply integrating sEMG features, gait features, and knee rehabilitation therapy, it establishes a closed-loop system progressing from “objective assessment” to “personalized intervention” and ultimately to “real-time feedback.” This approach holds significant implications for achieving early KOA identification, home-based precision rehabilitation, and enhanced long-term compliance. Guided by this overarching framework, this paper aims to:

1. Design a lightweight, wearable knee system for KOA patients, integrating multimodal sensing while ensuring daily wear comfort;
2. Propose a multimodal assessment method combining neuromuscular and joint kinematic and dynamic features to characterize patients’ “pain–function–fatigue” states during real-world activities;
3. Evaluate the system’s comprehensive effects on pain relief, gait features optimization, muscle fatigue delay, and subjective compliance to advance the development of a closed-loop, wearable, personalized intelligent rehabilitation system for KOA beyond existing diagnostic and rehabilitation technologies.

## 2. Materials and Methods

### 2.1. System Design

**Figure 1:**
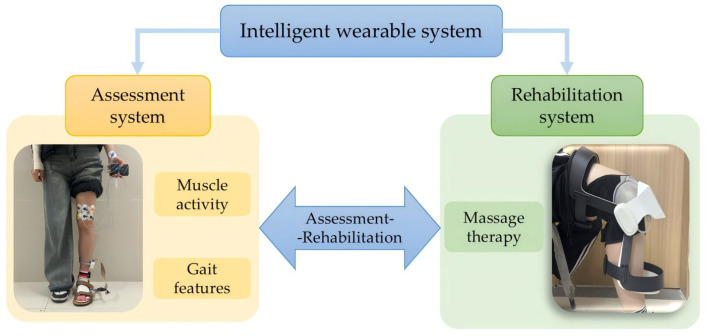
Design of the wearable system.

To address the aforementioned issues, this study proposes a wearable system for the assessment based on multimodal physiological signals and Intelligent rehabilitation of KOA. The system design is illustrated in Figure 1.

The system comprises two primary modules. First, the KOA assessment subsystem integrates multimodal sensors including sEMG signal acquisition devices, pressure sensors, and IMU. It continuously monitors and extracts lower limb muscle activity and gait features in real time, enabling a comprehensive evaluation of the patient’s knee joint functional status. Second, the knee brace-based massage therapy subsystem is based on the principles of the traditional Chinese medicine (TCM) “seated knee-adjustment method”, and it employs an airbag-driven mechanism to deliver automated mechanical intervention with controllable rhythms and adjustable intensity. This subsystem organically integrates traditional Chinese massage techniques with a wearable sensing system, providing technological support for intelligent KOA rehabilitation.

System design and prototype construction have been completed, with functional validation achieved for key modules.

### 2.2. Knee Osteoarthritis Assessment System

The KOA Assessment System serves as a core component of the overall framework, primarily responsible for acquiring, preprocessing, and extracting features from multimodal physiological signals. It provides data support for subsequent disease identification and feedback regulation in the rehabilitation module. This system can collect sEMG signals and kinematic data from patients in real time during movement. Through signal processing and feature analysis performed by the host computer, it extracts key physiological parameters reflecting the functional status of the knee joint.

**Figure 2:**
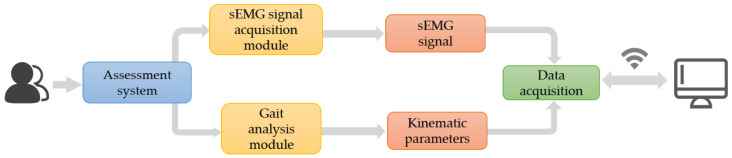
Design of the knee osteoarthritis assessment system.

As shown in Figure 2, the assessment system primarily consists of two components: the sEMG signal acquisition module and the gait analysis module that integrates pressure sensors and the IMU.

#### 2.2.1. Gait Analysis Module

Due to degenerative changes in the knee joint, patients with KOA exhibit abnormal gait parameters such as reduced walking speed, shortened stride length, and decreased knee flexion angle, resulting from pain and limited joint flexion and extension function [32,33]. Gait analysis measures and analyzes limb and joint movements during human walking, enabling quantitative measurement, description, and evaluation of gait features. Current gait features include kinematic metrics like stride length, cadence, ground contact time, and flight time. To enable comprehensive and portable gait analysis, this study employs a Force Sensitive Resistor (FSR) thin-film pressure sensor and IMU to construct a gait analysis module.

The FSR pressure sensor is a common type of force sensor, typically operating using a thin film made of one or more layers of conductive material. When external force is applied to the film, its resistance value changes, thereby measuring pressure [34]. This sensor offers advantages including low input force, light weight, high sensitivity, fast dynamic response, and repeatability.

**Figure 3:**
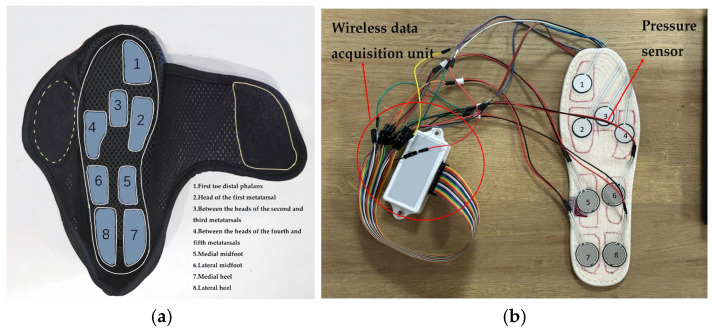
The pressure acquisition system. (**a**) The position of 8 pressure sensors; (**b**) Pressure sensors and wireless data acquisition unit.

During human movement, plantar pressure primarily concentrates at the first to fifth metatarsophalangeal joints and the heel region [35]. Therefore, this study selected pressure sensor positions corresponding to the first toe distal phalanx, the head of the first metatarsal, between the heads of the second and third metatarsals, between the heads of the fourth and fifth metatarsals, the medial and lateral midfoot, and the medial and lateral heel regions, as shown in Figure 3a.

This study employed FSR thin-film pressure sensors and GT-FSR thin-film pressure sensor data logger from Luoyang Guantop Electronics Technology Co., Ltd. (Luoyang, China) for pressure signal acquisition. The sensors featured a measurement range of 0–10 kg, a contact diameter of 16 mm, a thickness of 0.20 mm, a minimum measurable force of 500 g, a response time < 5 ms, and a hysteresis error of ±10%. According to literature reports, the peak pressure in the heel region and the second metatarsal region during natural gait is approximately 5.9 kg/cm^2^ [36], which translates to an applied force of about 7.28 kg. The selected sensor’s range fully covers this range. To achieve gait monitoring under natural conditions, the system employs a wireless data acquisition solution. The GT-FSR-12 data logger supports 12 synchronous input channels. This study utilizes 8 channels with a sampling frequency of 50 Hz. Data is transmitted to the host computer via Bluetooth BLE 5.2 protocol and stored in CSV format. The logger incorporates a built-in lithium-ion battery, ensuring continuous operation capability to meet long-term monitoring requirements in wearable scenarios. The pressure acquisition system is illustrated in Figure 3b.

IMU design principles primarily rely on measuring and analyzing an object’s acceleration and rotational motion [37]. By fusing data from accelerometers and gyroscopes, relatively accurate attitude information can be obtained. These are widely used in gait analysis and posture monitoring applications. This study systematically compared commonly used MPU-9250 (TDK InvenSense, San Jose, CA, USA), ICM-20948 (TDK InvenSense, San Jose, CA, USA), and industrial-grade IMUs during the selection process to meet the integration, power consumption, and real-time requirements of wearable gait monitoring systems. Industrial-grade IMUs offer higher measurement accuracy but feature larger dimensions and higher power consumption, making them unsuitable for the wearable application in this study. While the MPU-9250 and ICM-20948 exhibit lower power consumption and smaller size, they lack integration for wireless communication and power management. This necessitates additional Bluetooth modules and power management circuits, thereby increasing system complexity and power consumption.

Therefore, this study selected the IM948 IMU from Chenyi Electronic Technology Co., Ltd. (Huangshan, China). It integrates a three-axis accelerometer, gyroscope, and ARM 32-bit DSP processor, measuring 18.5 mm × 13 mm. It supports Bluetooth 5.3 BLE and serial communication, with a sampling rate of 0.5–250 Hz and an operating current of 7.6 mA. Key performance parameters include: acceleration range ±16 g (resolution 0.00048 g, measurement accuracy 0.01 g); angular velocity range ±2000°/s (resolution 0.061°/s, measurement accuracy 0.06°/s); in non-magnetic environments, *X*/*Y*-axis error is approximately 0.05°, and *Z*-axis error is approximately 0.1°. The system is powered by a lithium-ion battery. Through experimentation, the sensor orientation was determined as follows: the *X*-axis direction is parallel to the sagittal axis and aligned with the direction of travel; the *Y*-axis is parallel to the vertical axis; and the *Z*-axis is parallel to the coronal axis.

**Figure 4:**
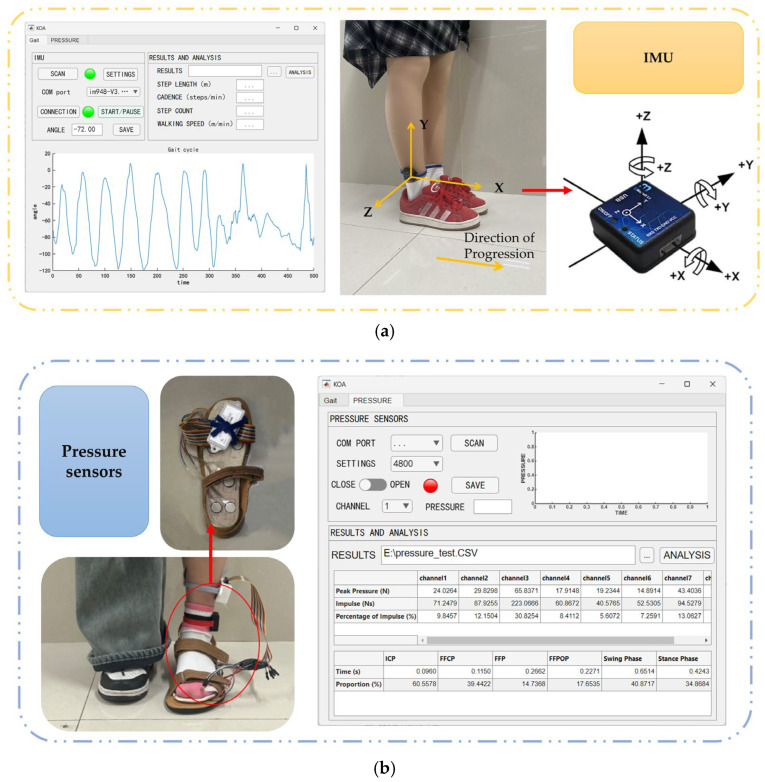
Schematic of the gait analysis module. (**a**) IMU sensor and its corresponding software interface; (**b**) Pressure sensor and corresponding software interface.

Our study employs MATLAB R2024a to construct a physiological parameter acquisition system. Eight pressure sensors are used to collect plantar pressure-related characteristics during patient walking, as shown in Figure 4a, while the IM948 module acquires kinematic parameters such as stride length and cadence, as shown in Figure 4b. During measurement, subjects wear the IMU on their ankles and utilize acquisition shoes equipped with built-in pressure sensors. To enhance system usability and clinical accessibility, the study specifically developed a visualization interface tailored for clinicians and researchers:

1. Provides device search, connection status indication, and data waveform display functions. It receives raw data in real time, decodes signals, and dynamically plots waveforms within callback functions. This enables operators to instantly monitor signal quality and acquisition stability, promptly identifying potential artifacts, drift, or connection anomalies;
2. Supports calculation of gait parameters from acquired data, graphically presenting postural changes throughout gait cycles. This provides intuitive evidence for identifying abnormal gait patterns, thereby enhancing the clinical interpretability of data and the timeliness and reliability of the evaluation process.

#### 2.2.2. sEMG Signals Acquisition Module

Patients with KOA often exhibit symptoms such as lower limb muscle weakness and joint instability, leading to impaired motor function. These changes can be captured through sEMG signals, which are primarily characterized by abnormal signals in aspects such as the activation sequence, activation degree, coordinated movement patterns, and fatigue patterns of lower limb muscles [38]. sEMG signals are bioelectric signals from neuromuscular activity recorded via electrodes on the skin surface. They correlate to varying degrees with muscle activity and functional status, thereby reflecting neuromuscular function to some extent. As the largest and strongest muscle in the lower limb forming the knee extensor mechanism, the structure and function of the quadriceps femoris muscle in relation to KOA pathogenesis are a hot topic in academic circles. The quadriceps femoris consists of four muscles: rectus femoris (RF), vastus medialis (VM), vastus lateralis (VL), and vastus intermedius (VI). Due to the difficulty of non-invasively measuring vastus intermedius sEMG signals, this study focused on sEMG from the RF, VM, VL [39].

**Figure 5:**
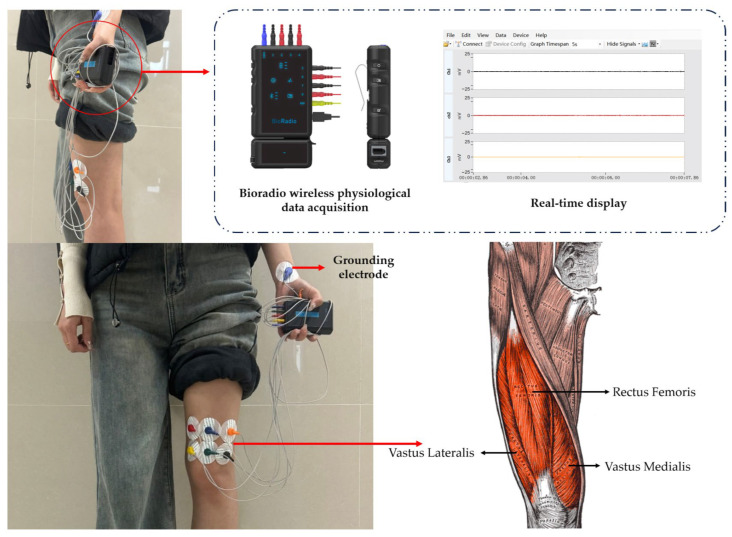
EMG Signal Acquisition Module.

sEMG signals from the patient’s quadriceps were measured using the BioRadio (Great Lakes NeuroTechnologies, Valley View, OH, USA) wireless physiological signal acquisition device. BioRadio is a portable, configurable physiological signal acquisition system capable of long-term, continuous collection and transmission of various physiological signal combinations without constraints. In this experiment, the electromyography signal sampling rate was set to 2000 Hz. The system employs a 24-bit ADC with a built-in 50 Hz notch filter to ensure high-resolution and low-noise signal quality. The main unit is powered by a lithium battery, enabling continuous operation for 8–12 h. Figure 5 shows the BioRadio device and its host computer software during the experiment. During the experiment, three pairs of differential electrodes were fixed at the following locations: The VM electrode was positioned at 20% of the distance from the medial joint space to the anterior superior iliac spine, with the line connecting the two electrode pads forming a 55° angle with the long axis of the femur. The RF electrode was placed on the anterior thigh, at the midpoint of the line connecting the upper border of the patella and the anterior superior iliac spine, with the electrode parallel to the long axis of the femur. The VL electrode was positioned above the lateral superior angle of the patella, at 10% of the distance from the lateral joint space to the anterior superior iliac spine, with the line connecting the two electrode pads forming a 15° angle with the long axis of the femur. The reference electrode was placed on the left wrist.

### 2.3. Knee Osteoarthritis Rehabilitation System

In China, TCM—encompassing acupuncture, moxibustion, herbal medicine, and massage—has been applied for thousands of years to treat various ailments [40]. Current research demonstrates that traditional therapies such as massage, acupuncture, and heat therapy yield verifiable efficacy in alleviating symptoms among patients with KOA [41]. The “seated knee-adjustment method”, as a TCM therapeutic method, has been extensively studied and shown to effectively alleviate pain and improve knee function in elderly KOA patients. This technique requires the patient to maintain a seated position with feet together and toes pointing forward, enabling the practitioner to stabilize the affected leg and accurately apply pressure with both thumbs to the depressions on either side of the patellar ligament. As the patient performs standing and sitting movements, the practitioner applies upward and inward pressure to adjust the joint axis and patellar position, thereby mobilizing the patellofemoral joint. Simultaneously, the practitioner’s other fingers encircle the popliteal fossa, further mobilizing the tibiofemoral joint through internal and external rotation of the tibia [42].

However, the number of practitioners proficient in this skill is limited, and its overuse can lead to finger injuries, posing significant occupational risks—particularly in healthcare settings with imbalanced physician-to-patient ratios. To meet clinical demands, the most mature solution involves using devices to simulate manual techniques, allowing machines to replace therapists in delivering treatment to patients.

To achieve intelligent implementation of this technique, this study designed a wearable intelligent rehabilitation system. The device must:

1. Apply precise force to the knee eye acupoint;
2. Continuously monitor dynamic changes in knee joint posture during the patient’s sitting-to-standing transition;
3. Be lightweight, low-noise, and comfortable for extended wear.

**Figure 6:**
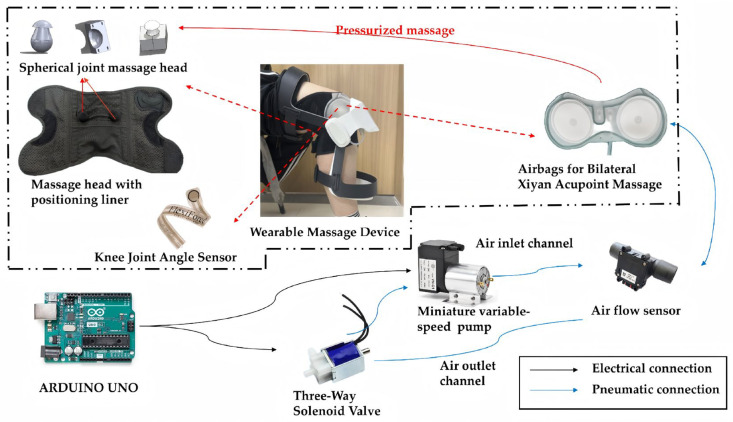
Knee Osteoarthritis Rehabilitation System.

To meet the aforementioned requirements, the system employs an airbag-pressurized system to drive spherical massage heads for force delivery, with the technical design system diagram shown in Figure 6. The spherical massage heads perform the massage actions, while the positioning liner ensures accurate targeting of the treatment area. The mobility of the spherical joints guarantees that the massage heads can track the movement of the massage site even when the patient’s posture changes. The airbag features a dual-layer structure: the lower layer envelops and stabilizes the knee while increasing gas volume for controllable massage force. The upper layer provides additional pressure and support, inflatable or deflatable as needed to accommodate different users.

**Figure 7:**
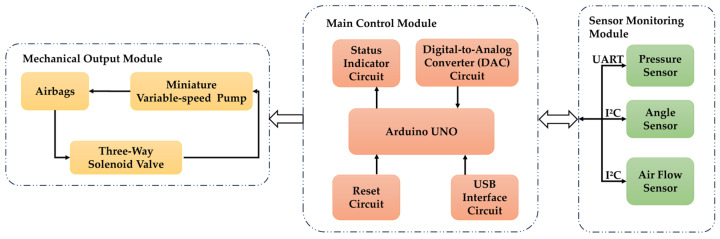
Control system of the device.

The drive system primarily consists of a mechanical output module and a sensor detection module. Figure 7 illustrates the control system of the massage device, with the Arduino UNO serving as the main control module responsible for coordinating the entire system’s operation. The core module of the mechanical output system comprises massage airbags, a miniature adjustable air pump, and a three-way solenoid valve, designed to perform the pressing and releasing actions characteristic of massage therapy. To meet the lightweight and low-noise requirements of wearable devices, a 20B miniature air pump (Zhejiang Kaimeng Mechanical Technology Co., Ltd., Hangzhou, China) was selected. Operating at a flow rate range of 0.5–1.2 L/min, it is driven by a brushless DC motor and features controllable rotational speed and overheat protection. Among these, the three-way solenoid valve and micro air pump, controlled by a microcontroller, switch between inflation and deflation processes. Different flow rate settings determine the volume changes in the dual-layer internal airbags, ultimately reflecting alterations in force applied to the patient’s knee eye acupoint. The sensor module comprises a Flexiforce thin-film pressure sensor (Tekscan, Norwood, MA, USA), a Flex Sensor4.5 angle sensor (Wuxi Sichiray Technology Co., Ltd., Wuxi, China), a flow sensor (Zhengzhou Winsen Electronics Technology Co., Ltd., Zhengzhou, China), and their respective converters. These components detect the patient’s reaction force applied to the massage head’s front end (i.e., the machine’s output massage force) and the bending degree of the built-in airbag (i.e., the patient’s knee joint flexion angle), enabling closed-loop monitoring and safety control of the treatment process. To ensure patient safety and therapeutic efficacy, the maximum output force is set to 50 N. The pressure sensor communicates with the main controller via Universal Asynchronous Receiver Transmitter (UART), while the angle and flow sensors transmit data using the Inter-Integrated Circuit (I^2^C) protocol. The flow sensor integrates an AC–DC conversion unit, directly outputting digital airflow sequences for real-time monitoring of airflow and airbag storage volume. These modules work together to form a closed-loop control system designed to facilitate rehabilitation for KOA patients.

### 2.4. Knee Osteoarthritis Assessment Features

#### 2.4.1. Selection of Gait Features

Gait features can be categorized into three major types: spatiotemporal features, kinematic features, and kinetic features. spatiotemporal features primarily describe gait rhythm and symmetry, including step speed, cadence, step length, stride length, step width, step angle, gait cycle, and gait phase. kinematic features reflect the spatial movement of key human body points or joints, primarily encompassing displacement, velocity, acceleration, joint angles, angular velocity, and angular acceleration. These metrics describe movement trajectories and joint coordination. kinetic features reveal the mechanical characteristics of gait, typically including plantar pressure distribution, ground reaction forces, joint moments, and center of gravity acceleration [43].

**Figure 8:**
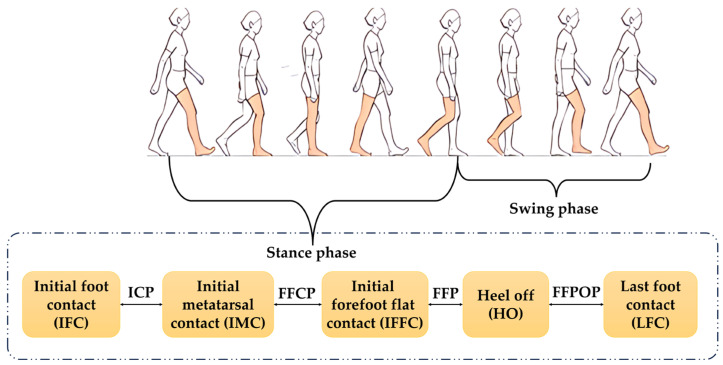
Division of the Gait Cycle.

In gait analysis, a complete gait cycle is defined as the process between two consecutive heel contacts on the same side, divided into the stance phase and swing phase. The stance phase is characterized by a sequence of five key gait events: Initial Heel Contact (IFC), Initial Metatarsal Contact (IMC), Initial Forefoot Flat Contact (IFFC), Heel Off (HO), and Last Foot Contact (LFC), as shown in Figure 8. Correspondingly, the single-leg stance phase is further subdivided into the Initial Contact Phase (ICP), Forefoot Contact Phase (FFCP), Flatfoot Phase (FFP), and Forefoot Push-off Phase (FFPOP) to reflect the dynamic changes in plantar loading and functional movement [44].

Research indicates that gait features of KOA patients exhibit significant differences compared to healthy individuals, with notable abnormalities in stride length, step length, walking speed, and the temporal proportions of heel contact and forefoot lift phases [32,45,46,47]. Therefore, this study selected the aforementioned key spatiotemporal and kinematic features as analytical indicators to quantify changes in gait patterns among KOA patients. Specific features are detailed in Table 1.

#### 2.4.2. Calculation of Gait Features

**Figure 9:**
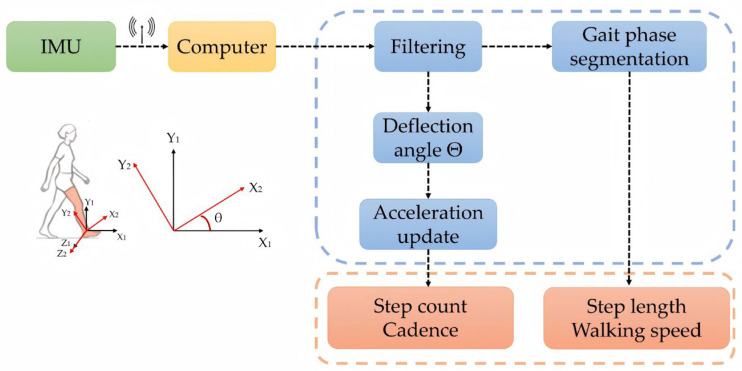
Workflow of IMU data processing.

Gait features such as cadence, step count, step length, step speed are derived from IMU data processing, with the processing workflow illustrated in Figure 9.

**Figure 10:**
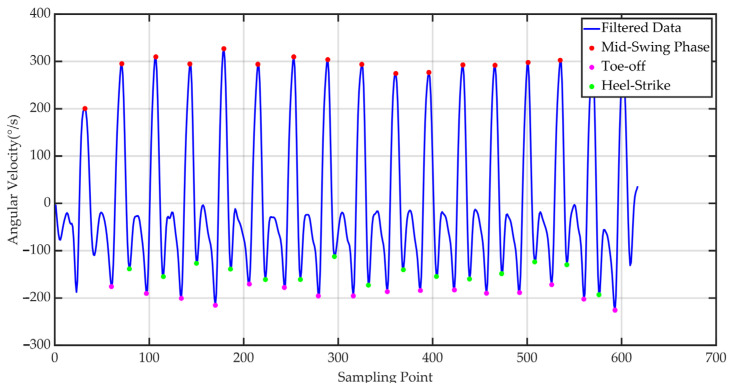
Gait cycle processing result.

To enhance gait event recognition accuracy, raw data undergoes preprocessing using a Butterworth bandpass filter. The filter cutoff frequency is set to 0.3–5 Hz to effectively suppress signal zero drift and high-frequency noise while preserving the primary frequency components within the walking cycle. Subsequently, based on the variation characteristics of acceleration and angular velocity signals, key event points within each gait cycle are identified, including toe-off, mid-swing, and heel strike, as shown in Figure 10.

Next, based on the quaternion attitude solution model, calculate the deflection angle θ of the lower limb during the gait cycle using Formula (1).

(1) $$\theta =arctan\frac{2\left(q_{1}q_{2}+q_{0}q_{3}\right)}{q_{0}^{2}+q_{1}^{2}-q_{2}^{2}-q_{3}^{2}}$$

then use this deflection angle to correct the acceleration component in the forward direction $a\left(t\right)$ using Formula (2).

(2) $$a\left(t\right)=a_{y}\left(t\right)sin\theta +a_{Z}\left(t\right)cos\theta$$

Furthermore, calculate the step length x, cadence f, and step speed v using Formulas (3)–(5).

(3) $$x=\int_{t_{i}}^{t_{i+1}}\left(\int_{t_{i}}^{t}a\left(\tau \right)d\tau \right)dt$$

(4) f=N/t

(5) v=x/t

**Figure 11:**
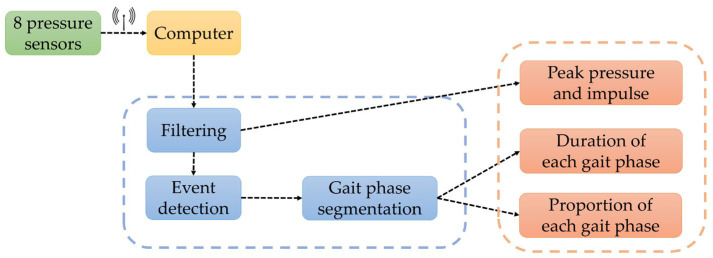
Workflow of pressure sensor data processing.

The remaining gait features were derived from pressure sensor data processing, with the workflow illustrated in Figure 11.

This study deployed eight pressure sensors across key load-bearing zones of the foot to capture real-time force variations during the gait cycle. The acquired pressure signals typically exhibit two distinct characteristics: first, periodic abrupt changes, where pressure signals show pronounced steep increases or decreases during foot contact or lift-off; second, local maxima, such as pressure peaks just before toe lift-off. During actual data acquisition, factors such as variations in the plantar contact surface, rebound from insole materials, and sensor vibration inevitably introduce short-duration high-frequency noise into the signals. While traditional filtering methods can partially remove this noise, they also weaken the abrupt edge transitions and smooth local maxima in the signals. This compromises the accuracy of gait event detection and temporal feature extraction, further affecting the precision of subsequent gait parameter calculations.

**Figure 12:**
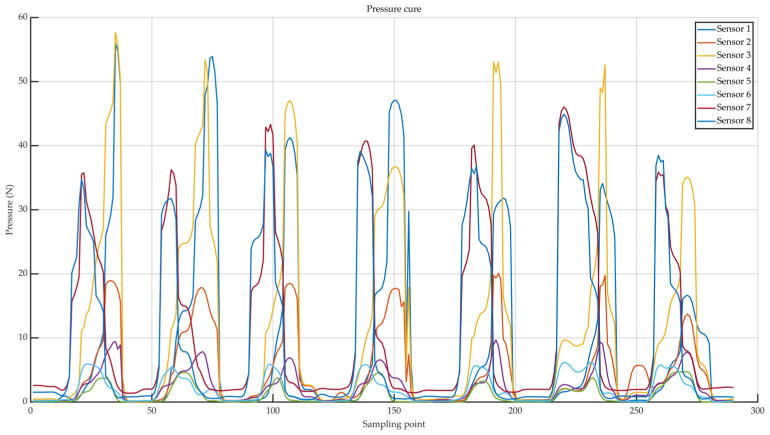
Multi-step plantar pressure waveforms.

Therefore, to enhance the smoothness and feature fidelity of plantar pressure signals, this study employs an adaptive weighted sliding filter for preprocessing the raw signals. This method dynamically adjusts weighting coefficients based on the amplitude fluctuations within local signal windows. During gradual signal changes, smoothing weights are increased to suppress high-frequency noise. When rapid signal variations or abrupt transitions occur, smoothing weights are reduced to preserve critical feature points. Compared to traditional fixed-window moving average filtering, this approach effectively reduces noise while preserving the integrity of critical gait event features such as pressure peaks and phase transition points. This provides higher signal quality for subsequent pressure peak detection and gait phase classification. Figure 12 shows multi-step plantar pressure waveforms collected during continuous walking. Each curve corresponds to one of eight pressure sensors embedded within the shoe. These sensors (Sensor1–Sensor8) are positioned at anatomical regions defined in Section 2.2.1 and illustrated in Figure 3a, including the first toe distal phalanx, the head of the first metatarsal, between the heads of the second and third metatarsals, between the heads of the fourth and fifth metatarsals, the medial and lateral midfoot, and the medial and lateral heel regions. Differences in amplitude and timing among sensor waveforms reflect characteristic loading patterns across these plantar regions during gait, such as foot contact and heel off.

Following filtering, pressure peaks and pressure impulses are calculated to quantify key force characteristics during gait. Pressure peaks reflect the maximum force exerted on specific plantar regions during a gait cycle, calculated using Formula (6),

(6) $$P_{peak}=max\left\{P_{1},P_{2},P_{3}\cdots P_{n}\right\}$$

Pressure impulse I characterizes the cumulative pressure change per unit time, calculated using Formula (7).

(7) $$I=\int_{t_{0}}^{t_{f}}p\left(t\right)dt\approx ∆t\sum_{k=1}^{n-1}\left(P_{k}+P_{k+1}\right)/2$$

**Figure 13:**
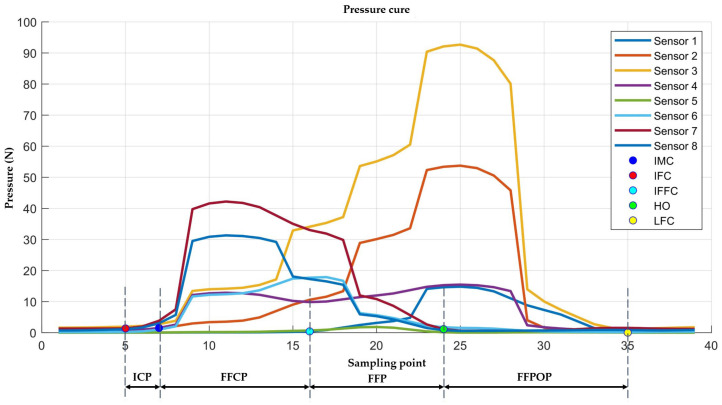
Key gait events identified result.

In Figure 13, a single gait cycle was extracted from the continuous plantar pressure signals, and the stance phase was segmented according to the gait-event definitions described in Section 2.4.1. Based on the five key event points (IFC, IMC, IFFC, HO, and LFC), the stance phase was further grouped into four functional sub-phases, including ICP, FFCP, FFP and FFPOP, as indicated in the figure.

Based on the time series of these key events, the duration of each gait phase is calculated using Formulas (8)–(11).

(8) $$T_{ICP}=t_{IMC}-t_{IFC}$$

(9) $$T_{FFCP}=t_{IFFC}-t_{IMC}$$

(10) $$T_{FFP}=t_{HO}-t_{IFFC}$$

(11) $$T_{FFPOP}=t_{LFC}-t_{HO}$$

The duration of the stance phase and swing phase are then determined using Formulas (12) and (13).

(12) $$T_{stance}^{n}=t_{LFC}^{n}-t_{IFC}^{n}$$

(13) $$T_{swing}^{n}=t_{IFC}^{n+1}-t_{LFC}^{n}$$

#### 2.4.3. Selection and Calculation of sEMG Features

The lower limb muscle activation patterns in KOA patients primarily manifest in two forms: First, high-level synergistic activation of multiple periarticular muscle groups, where multiple muscle groups exhibit strong, synchronized excitation during movement. This phenomenon results from compensatory activation due to degeneration and laxity of the musculoligamentous tissues surrounding the knee joint. Second, incomplete synchronous co-activation occurs between agonist, antagonist, and medial–lateral thigh muscle groups, reflecting an imbalance in the neuromuscular control system [48,49].

Additionally, KOA patients are prone to muscle fatigue accompanied by pain and joint structural degeneration. This not only significantly reduces daily activity levels but also triggers disuse atrophy in lower limb muscles. As muscle strength and joint stability further decline, patients experience earlier muscle fatigue during walking or weight-bearing, perpetuating a vicious cycle of “pain–disuse–strength loss–increased fatigue” [50,51,52].

Feature extraction from sEMG signals primarily employs three methods: time-domain analysis, frequency-domain analysis, and combined time-frequency analysis. In sEMG analysis, time-domain features like integrated electromyography (iEMG), zero-crossing rate (ZCR), variance (VAR) and root mean square (RMS) are commonly used to assess muscle activation intensity and fatigue levels [53]. Frequency-domain features of sEMG involve transforming the time-domain signal into the frequency domain via Fourier transform, followed by analysis of the power spectrum or spectral characteristics. The frequency-domain features selected for this study primarily include median frequency (MF) and mean power frequency (MPF), both of which sensitively reflect changes in muscle fiber conduction velocity and the progression of fatigue [54].

The raw sEMG signals acquired using the BioRadio system contain motion artifacts, baseline drift, and power-line interference. These noise components primarily arise from variations in electrode–skin impedance and environmental electromagnetic disturbances. Given that the physiological bandwidth of surface EMG signals typically ranges from 20 to 450 Hz, digital filtering was applied for signal conditioning. A band-pass filter (20–450 Hz) combined with a notch filter at the power-line frequency (50 Hz) is applied to remove low-frequency motion artifacts and baseline drift (<20 Hz), suppress high-frequency noise (>450 Hz), and attenuate power-line interference. The filtered signals provide improved signal quality, which enhances the accuracy of subsequent feature extraction and analysis.

**Figure 14:**
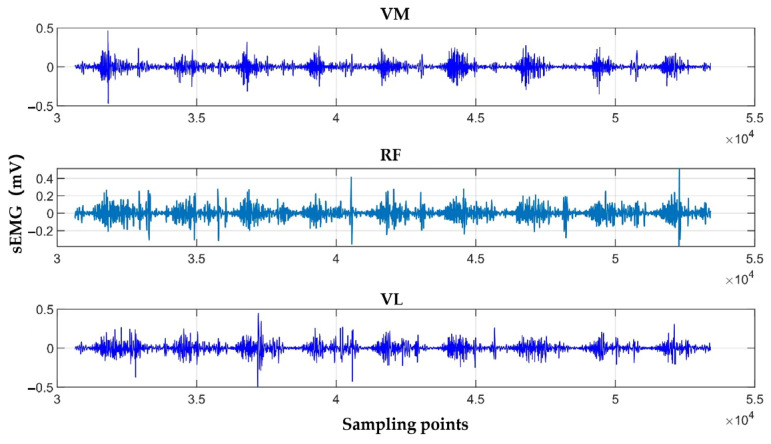
sEMG waveforms of the VM, VL, and RF muscles.

Figure 14 shows the sEMG waveforms of the VM, VL, and RF muscles after preprocessing and filtering.

Based on this, the time-domain characteristics (iEMG, ZCR, VAR, RMS) and frequency-domain characteristics (MF, MPF) of the sEMG for each muscle were calculated using Formulas (14)–(19), respectively, to quantitatively analyze the activation intensity and fatigue characteristics of the quadriceps femoris during walking.

(14) $$iEMG=\sum_{i=1}^{N}\left|x_{i}\right|$$

(15) $$RMS=\sqrt{\frac{1}{N}\sum_{i=1}^{N}x_{i}^{2}}$$

(16) $$ZCR=\sum_{i=1}^{N-1}sgn\left(-x_{i}x_{i-1}\right)$$

(17) $$VAR=\frac{\sum_{i=1}^{N}\left(x_{i}-\bar{x_{i}}\right)}{N}$$

(18) $$MPF=\frac{\int_{0}^{\infty }f\times PSD\left(f\right)df}{\int_{0}^{\infty }PSD\left(f\right)df}$$

(19) $$MF=\frac{1}{2}\int_{0}^{\infty }PSD\left(f\right)df$$

## 3. Results

### 3.1. Knee Osteoarthritis Assessment System Validation

#### 3.1.1. IMU Validation

To validate the accuracy of the gait feature calculation method proposed in this paper, five healthy subjects were selected as the control group. Participants wore IMU modules at designated locations as per experimental requirements and walked with a natural gait on an 18-m horizontal straight path. Simultaneous video recording during the experiment provided reference standard values for gait features (including stride length and cadence), which were compared with results calculated from sensor data, as shown in Table 2.

Relative Error (RE) was calculated to assess algorithm accuracy using Formula (20),

(20) $$RE=\frac{\left|Y-Y_{r}\right|}{Y_{r}}\times 100\%$$

where *Y* represents the calculated value and *Yr* represents the reference standard value.

Experimental results indicate that the average relative error for stride length is 7.79 ± 2.22%. Statistical analysis indicated no significant systematic bias between sensor-calculated values and reference values (*t* (4) = 0.548, *p* = 0.613). The two measures exhibited moderate correlation (Pearson *r* = 0.660, *p* = 0.226) and moderate consistency (Intraclass Correlation Coefficient, ICC (2, 1) = 0.630). The average relative error for cadence is 2.18 ± 0.43%, with no significant deviation detected (*t* (4) = 1.606, *p* = 0.184). The correlation is *r* = 0.702 (*p* = 0.186), and consistency is ICC (2, 1) = 0.600. This validates the high accuracy and stability of the designed system in IMU gait feature extraction.

#### 3.1.2. Pressure Sensor Validation

Since the pressure sensors in this study will ultimately be affixed to soft, deformable insoles, their force characteristics differ from those on rigid surfaces. Thin-film pressure sensors typically consist of a thin film, electrodes, and a support structure. When external force acts on the film surface, the film deforms, with the degree of deformation proportional to the magnitude of the external force. The strain distribution across the film differs between rigid and flexible surfaces. On a rigid surface, strain is concentrated primarily at the point of force application. In contrast, on a flexible surface, strain distribution is more uniform, encompassing both the force-applied area and surrounding regions. Therefore, to ensure measurement accuracy of the pressure sensor on a flexible insole, separate calibrations were performed under both rigid and flexible surface conditions.

**Figure 15:**
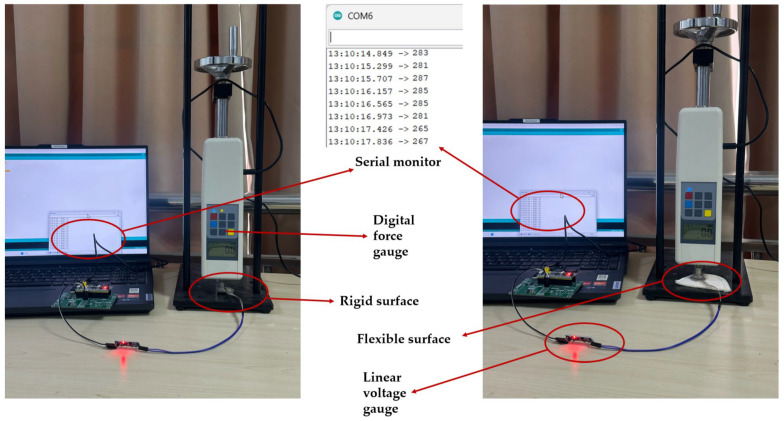
Experimental setup for the pressure sensor validation experiment.

During the experiment, the pressure sensor was fixed to the test platform. The rigid surface and the flexible surface are shown in Figure 15. A digital push-pull force gauge was used to sequentially apply pressures ranging from 10 N to 100 N (in 10 N increments) to the sensor. Each load was applied three times, and the output voltage values after passing through the voltage conversion module were recorded. The experimental data are recorded in Table 3.

**Figure 16:**
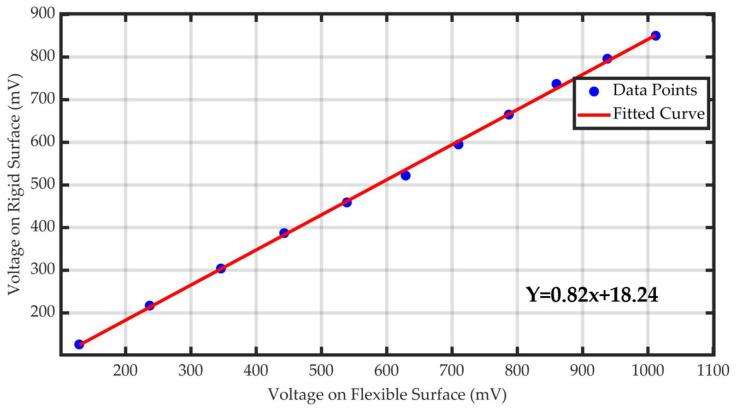
Fitting curves of the flexible and rigid surfaces.

To establish the pressure correspondence between the flexible and rigid surfaces, the average voltage measured on the flexible surface was plotted on the *X*-axis, and the average voltage on the rigid surface was plotted on the *Y*-axis. The data were imported into MATLAB for fitting, and the curve’s goodness-of-fit was calculated. The fitting results are shown in Figure 16. Furthermore, Table 4 presents the statistical metrics of the fitted models, including the estimated standard error (SEE), root mean square error (RMSE), coefficient of determination (R-square), and adjusted coefficient of determination (Adjusted R-square). Both the RMSE values are small, and the R-square and Adjusted R-square values are very close to 1. This indicates that the relationship between the pressure on the flexible surface and the pressure on the rigid surface can be well-fitted using a linear regression model.

### 3.2. Positioning Validation of the Rehabilitation System

#### 3.2.1. Massage Head Offset Error Experiment

**Figure 17:**
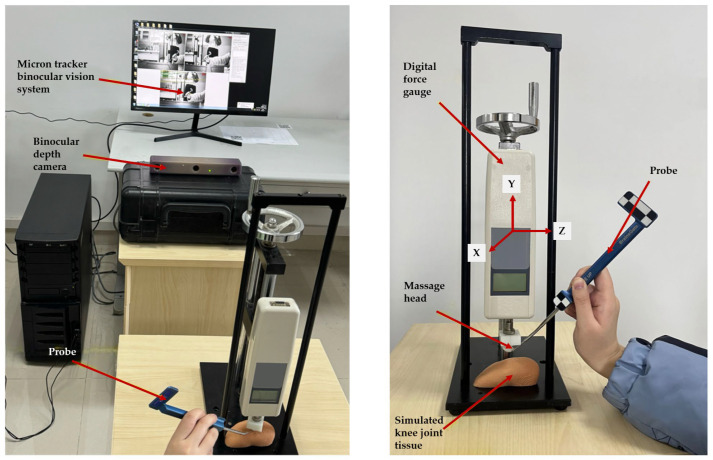
Experimental setup for the massage head offset error experiment.

To validate that axial force applied to the rear end of the massage head does not cause excessive drift of the front-end active massage head, this study employed a Micron Tracker binocular vision system and a digital push-pull force gauge to evaluate displacement errors generated by the ball-joint-based massage head during the massage process, as shown in Figure 17. A digital push-pull force gauge applied standard thrust to the rehabilitation system within the 0–70 N range, grouped in 5 N increments. During testing, the three-dimensional coordinates of the massage head’s apex were recorded throughout the force application process to obtain displacement data on the xoz plane, representing the offset error.

**Figure 18:**
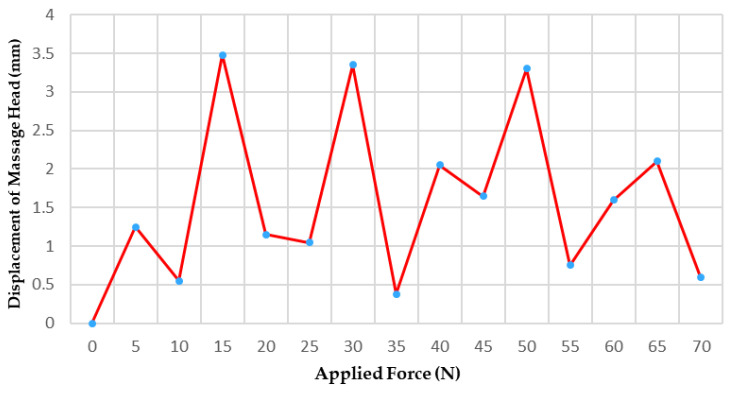
Displacement curve of the massage head in the offset error experiment.

Figure 18 shows the displacement curve of the massage head as a function of thrust. Experimental results indicate that the massage head exhibits displacement errors in the xoz plane, with a relatively small variation range. The displacement error spans 0.00–3.48 mm, averaging 1.55 ± 1.11 mm, with a 95% confidence interval of [0.93, 2.16] mm. Pearson correlation analysis revealed a correlation coefficient *r* = 0.172 (*p* > 0.05) between displacement error and applied force, indicating no significant correlation between the two variables. Further analysis indicates that the offset error of the massage head is primarily concentrated within the 1–3 mm range. Using the acupoint locations described in ‘’Nomenclature and Location of Acupuncture Point’’, this demonstrates that the proposed massage head design possesses satisfactory positioning accuracy and can meet clinical requirements.

#### 3.2.2. Massage Head Positioning Accuracy Experiment

**Figure 19:**
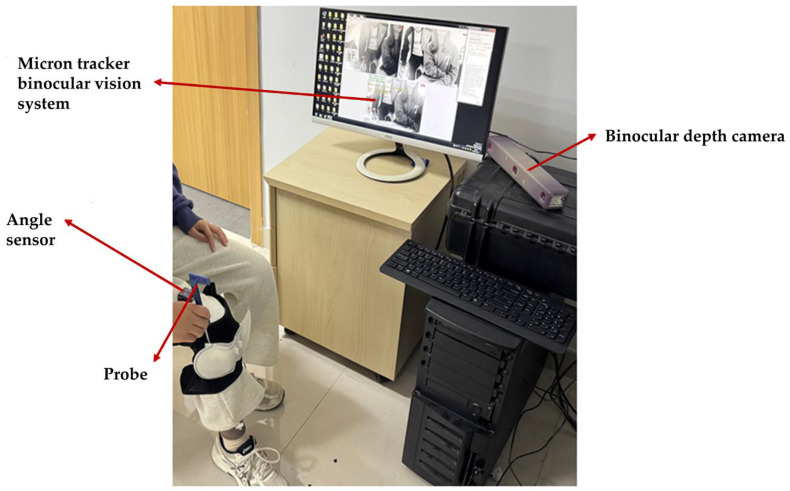
Experimental setup for the massage head positioning accuracy experiment.

To validate that the active massage head can track the displacement of the knee eye acupoint during patient posture changes, this study employed a Micron Tracker binocular vision system and angle sensors to measure the displacement of the massage head during the massage process, as shown in Figure 19. The sensor was fixed 8 cm above the patella on the subject, and the experimenter reminded the subject to perform the specified movements steadily throughout the process. After aligning the massage head with the knee eye acupoint, real-time massage therapy was administered in 10° increments per group. Between each group, the Micron Tracker binocular vision system identified probe markers to record the three-dimensional coordinates of the massage head’s vertex relative to the preset knee eye point in real time. By analyzing error data, the study calculated the massage head’s displacement in the xoz plane.

**Figure 20:**
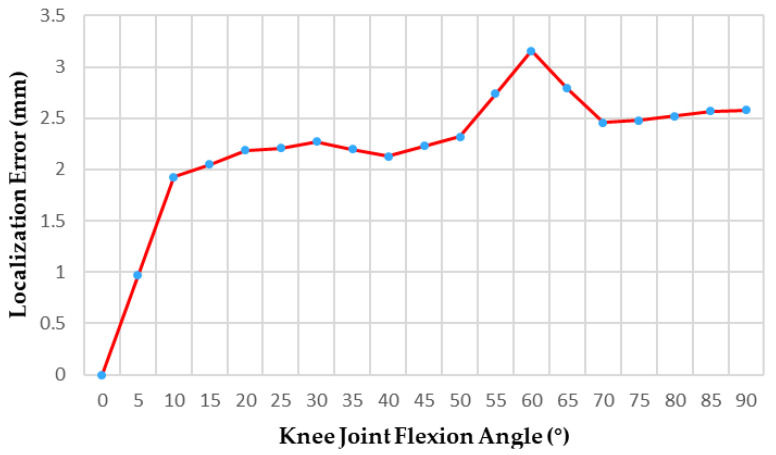
Displacement curve of the massage head in the positioning accuracy experiment.

Figure 20 displays the displacement curve of the massage head as a function of applied force. Analysis of the error data revealed an average positioning error of 2.20 ± 0.69 mm, with a standard error of 0.16 mm. The 95% confidence interval ranged from 1.89 to 2.51 mm, and the maximum deviation was 3.16 mm. The error distribution was stable, with no abnormal outliers, demonstrating high repeatability and reliability. Experimental results indicated that when the positioning liner effectively secured the massage head to the acupoint location, the spherical structure’s inherent mobility enabled effective tracking of the acupoint throughout displacement within the patient’s posture variation range (0° to 90°).

Additionally, the study incorporated a continuous experiment. Participants were instructed to wear the rehabilitation system while completing three seated-standing transitions, with the entire procedure repeated five times. Table 5 presents the error data collected across these five trials. The results indicate that in real-world working scenarios, cumulative errors occurred due to the massage head’s movement in response to knee joint angle changes. However, the overall error range remained relatively small, with the three-time average error controlled at approximately 1.80 ± 0.19 mm. Using the acupoint locations specified in the “’Nomenclature and Location of Acupuncture Point” as the positioning standard, the device’s positioning accuracy meets clinical requirements.

## 4. Discussion

This study addresses challenges in KOA management—including the large patient population, subjective clinical assessments, delayed imaging changes, difficulties in home rehabilitation, and the absence of a closed-loop evaluation-treatment-feedback system—by proposing and implementing a multimodal physiological signal-based knee function assessment and intelligent wearable rehabilitation system. The system enables real-time monitoring of patients’ knee “pain–function–fatigue” status during daily activities. Integrated with smart wearable therapeutic devices, it establishes a closed-loop management model spanning “objective quantitative assessment,” “personalized therapeutic intervention,” and “dynamic treatment feedback.” This approach offers novel engineering pathways and practical value for early precise identification of KOA, home-based personalized rehabilitation training, and long-term behavioral adherence improvement, demonstrating strong potential for clinical translation.

The overall system comprises two core submodules, one being the knee osteoarthritis assessment system. This system quantifies biomechanical characteristics closely associated with KOA pathological gait based on dimensions such as neuromuscular control, kinematics, and dynamics, enabling objective evaluation of knee joint function. Regarding gait feature selection, Fu et al. found that KOA patients minimize unilateral weight-bearing time on the affected side to reduce pain. Consequently, parameters such as heel contact time percentage, heel contact phase, full-foot contact phase, and forefoot lift-off phase exhibit significant differences compared to healthy individuals [55]. Elbaz A proposed a KOA gait classification method based on clustering and classification tree algorithms, identifying stride length and cadence as the most predictive indicators, achieving an overall classification accuracy of 89.5% (90.8% for females, 89.5% for males) [56]. These findings collectively indicate that KOA patients often modify gait rhythm and weight-bearing strategies to alleviate pain or reduce knee joint load. Significant differences exist between KOA patients and healthy individuals in stride length, stride frequency, walking speed, and the duration and proportion of each gait cycle phase, providing reliable evidence for selecting gait parameters in this study.

However, relying solely on gait features, while revealing macrokinetic abnormalities, lacks sensitivity for detecting functional degeneration in the early stages of the disease. Therefore, this study further incorporated sEMG signals from the quadriceps femoris muscle to supplement the assessment of knee neuromuscular function. Benedetti et al. found through simple EMG measurements that surface EMG signals from the quadriceps femoris muscle were lower in KOA patients compared to healthy individuals [57]. This indicates that EMG signals often show early changes in the initial stages of knee joint abnormalities, suggesting surface EMG signals can serve as an effective indicator for early KOA diagnosis. Luo et al. conducted a study on unilateral obesity-related KOA patients, using sEMG to quantitatively assess muscle activation and fatigue characteristics during isometric and dynamic knee movement tasks. Results revealed significant differences in muscle activation levels and fatigue indicators between the affected and unaffected sides [58]. Muscle function progressively deteriorated with increasing disease severity, further validating the feasibility of quantitatively assessing knee muscle function in KOA patients through electromyographic signaling systems. This system selected time-domain features (iEMG, ZCR, RMS) and frequency-domain features (MF, MPF) as core electromyographic features to quantitatively characterize the activation intensity and fatigue characteristics of the quadriceps femoris during walking, thereby achieving a more comprehensive and precise quantitative assessment of knee joint function.

Although this study has not yet undergone final clinical validation in KOA patients, performance evaluations of the IMU under laboratory conditions have demonstrated the system’s excellent measurement reliability. Experimental results show that the average relative error for stride length is 7.79 ± 2.22%, while the average relative error for cadence is 2.18 ± 0.43%, both maintaining high precision. Compared to the gait analysis system proposed by Harin Kim et al. based on the fusion of insole-mounted FSR and IMU (step length error ±7.17%, cadence error ±6.71%), the system in this study demonstrates superior stability and reliability in cadence calculation accuracy, further validating its feasibility for daily gait monitoring [59]. Furthermore, addressing the unique characteristics of pressure sensors on flexible surfaces, this study validated the accuracy of pressure measurement performance by fitting pressure response curves for both flexible and rigid surfaces. Based on statistical metrics including SEE, RMSE, R-square, and Adjusted R-square, the fitted model demonstrated excellent fit, indicating that pressure sensors can reliably capture pressure characteristics even when supported by flexible materials. These validation experiments establish a robust technical foundation for subsequent clinical studies targeting KOA patients. The next phase will focus on exploring correlations between these objective parameters and gold-standard clinical scales such as WOMAC and VAS, thereby further evaluating the system’s clinical potential as an assessment tool for KOA.

Another subsystem is the knee osteoarthritis rehabilitation system, which employs an airbag-driven mechanism to replicate the massage technique of the traditional Chinese medicine “seated knee-adjustment method” for targeted auxiliary treatment of patients. Current research has fully demonstrated the efficacy of this technique in KOA rehabilitation. Liang et al. reported in a randomized controlled trial involving 76 KOA patients that those receiving “seated knee-adjustment method” treatment demonstrated significant improvements in all WOMAC scale scores compared to pre-treatment levels [60]. Furthermore, after 4 weeks of treatment, patients exhibited more pronounced improvements in objective gait parameters such as stride length and step width (*p* < 0.05), comprehensively confirming its efficacy in improving knee function and enhancing walking ability. In the field of wearable therapeutic devices, Tomi Hakala proposed a compression sock design based on adjustable airbag pressure. This design not only enables effective control of pressure levels during prolonged wear but also accommodates user comfort requirements, providing reference for the controllable design of the airbag-driven mechanism in this study. A compression sock design utilizing adjustable-pressure airbags was proposed to achieve high controllability and comfort during prolonged wear [61]. Furthermore, extensive literature indicates that acupoint localization accuracy is closely correlated with the efficacy of traditional Chinese medicine [62,63]. Kang Zhiran [42], while elaborating on the operational essentials of the “seated knee-adjustment method”, explicitly stated that applying thumb pressure to the medial and lateral knee eye acupoint regions constitutes a critical component of this technique. Based on the aforementioned clinical and traditional medical evidence, and considering that the force application area of the proposed knee osteoarthritis rehabilitation system primarily involves spherical massage heads, experiments were conducted to evaluate massage head positioning deviation caused by two factors: airbag force application and patient posture changes. Experimental results indicate: under varying airbag force conditions, the average displacement error of the massage head was 1.55 ± 1.11 mm, with no significant correlation between displacement error and applied force magnitude (r = 0.172, *p* > 0.05). In tests simulating changes in patient knee joint angles, although the displacement error of the massage head showed a cumulative trend, the overall error remained small, with the average error over three trials controlled within 1.80 ± 0.19 mm. Compared to the acupoint location standards proposed in “Nomenclature and Location of Acupuncture Point”, the bionic massage head designed by the system achieves high positioning accuracy, sufficient to meet clinical requirements. However, the rehabilitation system proposed in this study has not yet undergone clinical validation. Future research will systematically evaluate functional changes before and after treatment using objective physiological indicators such as surface electromyography signals to comprehensively validate the system’s practical efficacy in KOA rehabilitation.

Compared to traditional methods for assessing KOA, the system proposed in this study enables a more comprehensive and quantitative analysis of knee function. Leveraging wearable and intelligent design, it significantly enhances usability and compliance for patients in home rehabilitation settings. Although the system demonstrates feasibility in engineering implementation and functional integration, several limitations remain: First, current physiological features are based solely on individual data, lacking sufficient population-based reference standards, which may compromise the accuracy of disease progression assessment. Second, the rehabilitation module has yet to implement personalized treatment strategies tailored to individual differences. Third, the system has not established a unified mapping relationship with clinical gold standard quantitative metrics.

To further enhance the clinical utility of the system, future research will focus on the following areas:

1. Based on the engineering validation results from this study, a clear plan for subsequent clinical research has been established. The next step involves recruiting patients diagnosed with KOA according to the K-L classification. While using this system to collect multimodal physiological signals, patient-reported outcome measures including WOMAC and VAS will be recorded. Correlation analysis and regression modeling will be employed to construct a mapping model between multimodal physiological signals and clinical scores;
2. Combining expert massage techniques with patient physiological characteristics (e.g., BMI) to establish personalized rehabilitation parameter adjustment models, enabling adaptive massage force output driven by airbags;
3. Establishing standardized clinical evaluation and real-time feedback mechanisms to form a closed-loop rehabilitation process encompassing “objective detection—intelligent analysis—personalized intervention—outcome feedback,” thereby further enhancing the system’s reliability and clinical translation potential.

In summary, the multimodal physiological signal assessment and intelligent rehabilitation system developed in this study provides a feasible and scalable technical pathway for the objective evaluation and home-based intervention of KOA, laying the foundation for future intelligent diagnosis and treatment of KOA.

## Figures and Tables

**Table 1:** Selected gait features.

Sensor	Features
IMU	Cadence, Step count
	Step speed, Step length
Pressure sensor	Impulse, Proportion of Impulse
	Peak Pressure
	Duration of the Stance/Swing Phase
	Proportion of the Stance/Swing Phase
	Duration of the ICP/FFCP/FFP/FFPOP
	Proportion of the ICP/FFCP/FFP/FFPOP

**Table 2:** Results of the IMU sensor validation experiment.

Group	Step Count			Stride Length			Cadence		
	Measured Value (Steps)	Calculated Value (Steps)	Error (Steps)	Measured Value (m)	Calculated Value (m)	RE (%)	Measured Value (Steps/min)	Calculated Value (Steps/min)	RE (%)
1	16	16	0	1.13	1.02	9.73	98	100	2.04
2	15	16	1	1.20	1.31	9.17	105	103	1.90
3	18	18	0	1.00	1.09	9.00	99	101	2.02
4	16	16	1	1.13	1.08	4.42	102	105	2.94
5	15	14	1	1.20	1.28	6.67	101	103	1.98

**Table 3:** Results of the pressure sensor validation experiment.

Surface Material	Applied Force (N)	Voltage After First Loading (mV)	Voltage After Second Loading (mV)	Voltage After Third Loading (mV)	Average Voltage (mV)
Rigid Surface	0	126	131	122	126
	10	223	220	210	217
	20	314	302	297	304
	30	395	379	388	387
	40	466	452	461	459
	50	527	515	524	522
	60	600	591	596	595
	70	670	663	663	665
	80	737	731	744	737
	90	803	790	795	796
	100	859	846	845	850
Flexible Surface	0	128	129	131	129
	10	236	240	235	237
	20	343	352	343	346
	30	442	445	443	443
	40	549	535	534	539
	50	632	625	630	629
	60	713	708	710	710
	70	786	791	784	787
	80	858	865	857	860
	90	937	941	935	938
	100	1009	1010	1018	1012

**Table 4:** Curve fitting performance results.

Goodness of Fit	
R-square	0.99922
Adjusted R-square	0.99913
RMSE	6.3982

**Table 5:** Results of the continuous positioning accuracy test.

Test Round	Positioning Error During the First Seated-to-Standing Transition (mm)	Positioning Error During the Second Seated-to-Standing Transition (mm)	Positioning Error During the Third Seated-to-Standing Transition (mm)
1	1.32	1.69	2.08
2	0.85	1.36	1.70
3	1.22	1.57	1.89
4	0.93	1.48	1.77
5	1.04	1.19	1.58
Mean ± SD	1.07 ± 0.20	1.46 ± 0.19	1.80 ± 0.19

## Data Availability

The original contributions presented in this study are included in the article. Further inquiries can be directed to the corresponding author.
